# Exit, voice or neglect: Understanding the choices faced by doctors experiencing barriers to leading health system change through the case of Sierra Leone

**DOI:** 10.1016/j.ssmqr.2022.100123

**Published:** 2022-12

**Authors:** Oliver Johnson, Foday Sahr, Nick Sevdalis, Ann H. Kelly

**Affiliations:** a Centre for Implementation Science, Health Services & Population Research Department, Institute of Psychiatry, Psychology & Neuroscience, King's College London, London, United Kingdom; b Centre for Health Policy, School of Public Health, University of Witwatersrand, Johannesburg, South Africa; c Department of Microbiology, College of Medicine and Allied Health Sciences, University of Sierra Leone, Freetown, Sierra Leone; d Military Hospital, Wilberforce, Sierra Leone; e Department of Global Health & Social Medicine, School of Global Affairs, Faculty of Social Science & Public Policy, King's College London, London, United Kingdom

**Keywords:** Leadership, Leadership development, Clinical leadership, Hirschman, Sierra Leone, Sub-Saharan Africa

## Abstract

This paper presents a study from Sierra Leone that explored the experiences of doctors as they endeavored to improve the health care systems in which they worked. Twenty-eight interviews were conducted with doctors in Sierra Leone, complemented by long-standing experience of national health provision and research by the authors. Drawing on Hirschman's theory of ‘*exit, voice and loyalty*’, the paper's framework analysis elaborates the doctor's career decisions and choices under systematic political and economic constraints, and in particular, the specter of retribution, including posting to undesirable jobs and withholding of salaries. This retribution was considered a driver of exit by doctors from the system, and few examples were given of doctors successfully advocating for change through advocacy (‘voice’). We suggest that the relevance of Hirschman's theory to this setting is in drawing attention to the critical themes of retribution, opportunity, loyalties, and partial exits, ones often neglected in efforts to reduce emigration of doctors and strengthen their leadership. Ultimately, this paper critiques the overemphasis of mechanistic ‘capacity building’ in global health and recommends that health system strengthening must be viewed as a jointly political as well as technical exercise.

## Highlights

• Leadership is a key building block in any health system.
• There have been few studies on barriers to doctors leading change.
• Doctors can experience retribution resulting from efforts to lead.
• Hirschman's theory illuminates how it drives doctors to emigrate or be silenced.
• Health systems strengthening is political as well as a technical exercise.

## 1 Introduction

Widely recognized as key to the delivery of effective health services, the World Health Organization (WHO) has described leadership as “arguably the most complex but critical building block of any health system” (2007b, p. 23). Definitions of leadership are contested and wide-ranging, but in the context of health policy, a critical component involves driving improvement and change (Heifetz, 1994). Given their “frequent role as head of the healthcare team and commander of considerable clinical resources” (NHS, 2010, p. 5), doctors play a particular significant role in those efforts. Understanding the choices available to doctors experiencing barriers to leading change, in particular barriers that may damage or derail the doctor's career, are critical to health systems strengthening (Creese et al., 2021). As the WHO notes, leadership involves “both political and technical action, because it involves reconciling competing demands for limited resources, in changing circumstances” (World Health Organization, 2007a, p. 23).

This study draws on the experiences of doctors in one Sub-Saharan African country, Sierra Leone, to explore these dynamics. We build on a recent qualitative study on leadership by doctors in Sierra Leone, where participants described relatively few examples of positive change in the health system, which sometimes required strike action to be achieved. The study documented significant barriers experienced when trying to lead change, illustrated well by this quote reported in the study: *“Sometimes you face threats, threats of either losing your job, threats of being victimized in the system as a junior doctor”* (Johnson et al., 2021)*.* A separate qualitative study of medical student experiences in Sierra Leone revealed similar threats and favoritism in undergraduate education (Woodward & McLernon-Billows, 2018). We explore how these threats impact not only on health system reforms but also the retention of health workers within the system or their employing organization. In their systematic review on retention of health workers in low and middle income countries, Willis-Shattuck et al. (2008) found that 80% of studies identified poor health management as a driver of emigration and low motivation, with health workers commenting that “their supervisor's management and leadership skills were inadequate” and that they often failed to lobby on behalf of their team.

We consider the leadership roles of doctors practicing medicine and attempting to lead change within the country's healthcare system; but also the ongoing roles in the Sierra Leonean health system of doctors who have emigrated and are in the global ‘diaspora’, which we define as “first generation migrants … [who are] sympathetic to the development of their ancestral homeland” (Page & Mercer, 2018).

Sierra Leone provides a compelling context for this enquiry. In Sierra Leone, health outcomes are poor and the shortage of doctors is deemed critical, in significant part due to emigration (World Health Organization, 2020). The country's recent experience with a major Ebola outbreak, when doctors faced extreme personal risk alongside substantial organizational upheaval and political interference (Tengbeh et al., 2018; Walsh & Johnson, 2018), can be illustrative of health systems worldwide that have been affected by the ongoing coronavirus-19 (COVID-19) pandemic.

To guide us in identifying the choices faced by Sierra Leonean doctors experiencing barriers to leading change, we draw on the work of Albert O Hirschman, who theorized the choice faced by individuals who find themselves in failing organizations: to leave in protest (‘exit’) or to stay and advocate for change (‘voice’) (1970). The development of this theory has its roots in the study of the public railway system in Nigeria and it has been used in the analysis of contexts as diverse as salaries in the public workforce and dissatisfaction in romantic relationships (Lee & Whitford, 2008; Rusbult et al., 1982).

In using Hirshmann's theory to think through the moral negotiations and thorny decisions taken by doctors, this paper departs from conventional strands in the health system literature that focus on discrete medical capabilities and consider health system strengthening to be primarily a matter of ‘capacity building’ – a rather technical, if not mechanistic, perspective. While instructive, without addressing the broader contexts needed to effect positive change, in particular endogenous change, efforts at reform may be rendered superficial at best. This paper posits that health systems strengthening should be viewed as a political as well as a technical process, requiring attention to understanding and removing inhibitors of domestically-driven reform and the cultivation, first and foremost, of loyalty.

## 2 Exit, diaspora

Hirschman's theory is concerned with change, specifically how any kind of organization, be it a private company or a national government, can be stimulated to improve or correct a lapse in performance through the actions of customers or staff, who can choose ‘exit’, ‘voice’ or ‘loyalty’. To ‘exit’ means to leave an organization in protest, such as by switching purchases to a competitor firm or resigning from a job; while some people may exit quietly, others may attempt a ‘noisy exit’, for example by publicizing their concerns in the media or elsewhere (Withey & Cooper, 1989). Alternatively, Hirschman describes ‘voice’ as “any attempt at all to change, rather than to escape from, an objectionable state of affairs,” such as by “changing the practices, policies, and outputs of the firm from which one buys or of the organization to which one belongs'’ (Hirschman, 1970, p. 30). The final component of the theory is ‘loyalty’, the ties that bind an individual to an organization. Hirschman primarily positions loyalty as a moderating factor that can influence the decision to use exit or voice, arguing that “loyalty holds exit at bay and activates voice” (Hirschman, 1970, p. 78). In some instances, however, Hirschman frames loyalty as an alternative to exit and voice, a situation whereby individuals “simply refuse to exit and suffer in silence, confident that things will soon get better” (Hirschman, 1970, p. 38).

Hirschman's theory has proved to be highly versatile - offering a conceptual latitude that we believe is well suited to framing the complex choices faced by doctors. The empirical prompt for our analysis is encountering barriers or retribution to efforts to lead change. By empirical prompt we mean the negative experience of a doctor that might cause them to consider voice or exit. Doctors may ‘exit’ by moving from the government to the private sector or by emigrating abroad. Alternatively, they may use ‘voice’ to continue advocating for change through their roles in the system or through advocacy or strike action. Finally, they may opt to turn a blind eye to failings such as corruption or dangerous patient care, or become complicit in those activities in order to advance their own interests.

We are not the first to apply Hirschman's theory to an analysis of the health sector. While most commonly deployed to explore the relationship between patients and care providers (James & John, 2021; Pickard et al., 2006) the voice, loyalty, exit framework has helped to illuminate the dynamics between health professionals and their employers. Lachman and Noy (1996) for instance, drew upon Hirshmann to examine the reactions of salaried physicians to hospital decline in the USA, while more recently two studies applied his analysis to the emigration of doctors out of Ireland (Creese et al., 2021; Humphries et al., 2019). In the context of global health, Chesang (2013) has used the framework in a Sub-Saharan African setting to understand a range of national and household dynamics in Kenya, including health care, while Zweigenthal et al. (2018) applied it to inform decisions by doctors in South Africa to specialize in public health.

In what follows, we extend this work by examining the decisions of doctors experiencing barriers to change. A particular focus of our analysis is on the role of health workers in the diaspora, who we suggest have exited but continue to attempt to use voice from abroad. Central to this decision to exit but remain involved in the health system is loyalty, which we explore in some detail. Finally, we examine the characteristics of the health system that may make it more or less sensitive to exit and voice.

### 2.1 Context

Sierra Leone's only medical school, the College of Medicine & Allied Health Sciences (COMAHS), opened in 1988 and most postgraduate training still takes place outside of the country, although a Postgraduate College of Health Specialties was formally launched in 2021 (Government of Sierra Leone, 2021). The large majority of new Sierra Leonean doctors now train at COMAHS, which currently graduates approximately fifty medical students per year, of whom almost all are nationals, as opposed to foreign students. Despite this, the country only had 156 junior and middle grade doctors and 41 medical specialists in 2016, demonstrating very high levels of medical professional emigration and a critical shortage of health workers (Ministry of Health and Sanitation Government of Sierra Leone, 2016, pp. 1–71). There are no recent studies with specific data on health worker emigration from Sierra Leone, although a report on staffing in the public health sector from 2005–11 found attrition rates of doctors averaging as 13% for medical officers, 41% for registrar doctors and 33% for consultants, where attrition could be from a range of causes including emigration, retirement or death (Wurie et al., 2014).

Doctors generally either follow a clinical pathway, working as medical officers or specializing and taking consultant posts, or they study public health and take on health management roles. Most government health institutions are led by doctors including secondary and tertiary hospitals, district health management teams and many Ministry of Health & Sanitation (MoHS) departments. Doctors commonly run their own private clinics alongside roles in government hospitals, and many senior doctors hold multiple posts including at the University of Sierra Leone and MoHS.

Sierra Leone experienced a major outbreak of Ebola from 2014 to 2016, which led to significant loss of life amongst health professionals and disruption to their training (Tengbeh et al., 2018). That outbreak extenuated the circumstances of a highly extractive colonial rule that ended in 1961. This was followed by nearly two decades of authoritarian leadership and subsequently a civil war which ended in 2002, which had a profound impact on the country's economy and workforce, with many professionals emigrating from the country. Sierra Leone has since experienced multi-party democracy and economic growth, although levels of unemployment, poverty and corruption remain high (Reno, 1995; Walsh & Johnson, 2018).

## 3 Methods

This paper develops from an earlier qualitative study of leadership by doctors in Sierra Leone, which explored perceptions of effective leadership and the contextual challenges experienced by doctors trying to lead Johnson et al. (2021). A key finding of that work was that doctors faced retribution from their leadership efforts. Using in-depth interviews, this study sought to develop the issue further by examining the impacts that retribution has on doctors’ career decisions and subsequent efforts to push for change.

### 3.1 Study design

Twenty-eight interviews were conducted in total, split into two phases. In the first phase, which took place between October 2019 and February 2020, fourteen exploratory interviews were conducted, that investigated perceptions of leadership by doctors in general. In the second phase, a further fourteen interviews were conducted between April and July 2021 that focused specifically on barriers to leading change.

### 3.2 Role of the researchers

All interviews were conducted by the first author, who is a medical doctor from the UK who had previously spent three years working within the government health sector in Sierra Leone. As such he had prior relationships with many doctors in the country and some understanding of contextual issues mentioned in the interviews such as events, places, and political dynamics. The second author is a Sierra Leonean doctor and senior academic with extensive knowledge of people and context, while the last authors are UK-based academics with experience in psychology, patient safety and anthropology that have previously collaborated on research in Sierra leone.

### 3.3 Participant selection

Participants from the medical profession were identified through a combined snowballing and purposive sampling framework, informed by the prior experience of the authors in the country's health system. This included representation from public and private sectors, different seniorities, varied career pathways, and inclusion of doctors who had emigrated from the country. We attempted to mitigate the potential biases introduced by this in-group approach, by intentionally including diverse perspectives and made this explicit when seeking advice on recruiting additional participants (Noy, 2008). Furthermore, confidentiality was emphasized and a significant number of participants were identified who had no previous relationship with the interviewer. Participation was voluntary, with no incentives offered.

### 3.4 Interviews & sample size

Most interviews were conducted online using videoconferencing software, but some initial interviews were conducted in-person in private spaces. Topic guides were used to orient discussions and the topic guide for the second phase of the interviews is included in Supplementary Appendix A. The topic guides were developed by the authors and piloted with a single interview, after which no adjustments were required. The second phase of the interviews specifically explored participants' perceptions of how Hirschman's theory applied to barriers to leading change that they had experienced or observed in Sierra Leone, how doctors responded to these. ‘Exit’ was defined as ‘people leaving the country or the government health sector’. ‘Voice’ was described as ‘deciding to stay in the government health sector and advocate for change’. The concept of ‘leadership’ was left undefined since it was the term for which we were seeking empirical elaboration, and participants were invited to define ‘loyalty’ themselves since the concept is poorly explained in Hirschman's original theory. Interviews were between 23 and 125 ​min in length and were conducted in English.

The sample size of each phase of fourteen interviews was calculated using existing methods, which demonstrate that saturation can be reached by twelve interviews (Guest et al., 2006). For our second phase of interviews, saturation was reached after twelve interviews. Interviews were audio recorded and transcribed verbatim using Nvivo 12 software (InternationalQSR, 2018).

### 3.5 Data analysis & development of themes

Each transcript was reviewed in detail and codes assigned to sections of data by the lead author. Initial codes developed by the lead author were reviewed by two other authors and feedback given. A framework approach to thematic coding was adopted (Ritchie & Spencer, 1994). The three primary components of Hirschman's theory, ‘exit, ‘voice’ and ‘loyalty’, were used to initially code the data deductively. Additional themes were then identified inductively based on the specific experiences described by participants. Initial themes were developed by the lead author and presented for discussion from other authors through several rounds of review. This enabled the development of a graphical representation of an expanded version of Hirschman's theory to present the choices available to doctors facing barriers to leading change. The characteristics of our participants are summarized in Table 1, divided into the first and second phases of the research.

**Table 1:** Characteristics of the study participants.

Characteristic		Phase 1	Phase 2	Total
**Gender**^a^	Female	3 (21%)	5 (36%)	8 (29%)
	Male	11 (79%)	9 (64%)	20 (71%)
**Seniority**	Junior	4 (29%)	2 (14%)	6 (21%)
	Middle-Grade	2 (14%)	4 (29%)	6 (21%)
	Senior	8 (57%)	8 (57%)	16 (57%)
**Location**^b^	Sierra Leone (public)	11 (79%)	8 (57%)	19 (68%)
	Sierra Leone (private)	1 (7%)	2 (14%)	3 (11%)
	Abroad (temporary)	2 (14%)	1 (7%)	3 (11%)
	Abroad (permanent)	0 (0%)	3 (21%)	3 (11%)
**Career Pathway**^b^	Clinical	9 (64%)	9 (64%)	18 (64%)
	Public Health	2 (14%)	5 (36%)	7 (25%)
	Academic	3 (21%)	0 (0%)	3 (11%)

a In 2016, 18% of doctors in Sierra Leone identified as women and 82% as men (Ministry of Health and Sanitation Government of Sierra Leone, 2016, pp. 1–71).

b As many participants held multiple different roles, we selected a primary role for each participant.

### 3.6 Ethics

Ethical approval was granted by the Sierra Leone Ethics & Scientific Review Committee and by King's College London. All participants provided written informed consent prior to the interview conduct.

## 4 Findings: voice, loyalty, exit

In presenting our findings, we begin with an exploration of the key barriers to leading change experienced by doctors in Sierra Leone. We then examine the role and impact of ‘exit’ resulting from these challenges, and how opportunity to leave can moderate this decision. We go on to discuss the effectiveness of staying in the government health system by adopting ‘voice’ or ‘neglect’, including the ways that loyalties can shape these decisions. Finally, we summarize the perceived relative effectiveness of exit and voice in triggering change in the health system.

In addition to the quotes included in the text of the Results section, additional qualitative data have been set out in the Supplementary Appendix B, categorized by section.

### 4.1 Retribution as tradition

The concept of leading change is understood here quite broadly as any effort to alter the status quo with the intention of bringing positive benefits to the health system and to patients using it. In some instances, this might be through formal changes, such as re-designing a process or advocating for improvements in salaries or equipment. In other occasions, the focus might be informal and involve resisting pressures to behave inappropriately, such as refusing to demand unofficial payments from patients or to alter the marks of politically connected students. Of interest to this study was leadership, which we defined as ‘mobilizing people to tackle tough problems’ (Heifetz, 1994). Our emphasis was therefore on the more challenging efforts of leading change that generated resistance, which was common, and which often resulted in retribution against the doctor leading it.

Participants consistently described a set of negative personal or professional consequences that were commonly experienced by doctors in Sierra Leone trying to lead change. While these consequences were most often orchestrated by senior officials, they could also be driven by colleagues or the public.

The most prominent negative consequence was the reposting of doctors by senior MoHS officials to remote or otherwise undesirable or dangerous postings. Participants described how it was routine and common knowledge that doctors who challenged or upset senior officials could expect to be reposted at the next opportunity.

*“The punitive action that is usually taken against young colleagues … when they try as best as possible not to bow down to the usual corruption or state that you usually see in some of these hospitals, is for them to be posted to the rural province. And the rural province is basically like a yardstick that is being used to punish young doctors, because we know everybody wants to stay in Freetown.”* Senior doctor, male

Other forms of bureaucratic retribution included the withholding of salaries and the withdrawal of scholarships for international postgraduate training.

*“So, after some time, [a senior official] called me to his office … he sat me down and said ‘ … You have to respect people here irrespective of whatever happens. Your bosses here, they told me you are very disrespectful, and I have even been instructed that I should ensure that you are not paid salary for summer months.’”* Middle-grade doctor, male

Doctors who challenged authority could also lose their jobs or have their careers stalled. Such consequences could also come from juniors or peers who might try to sabotage a person's career for threatening their interests.

*“[The senior officials'] power was absolute. And so this guy we wanted to hire, they straight up told [an international organization] ‘You will not hire him, we will not release him. He’s not hirable’. And I was like, ‘What do you mean, this is the most qualified person for the job?‘ … They were this adamant, the vendetta was so strong against this guy.”* Senior doctor, female

Participants also described being cold-shouldered by the MoHS, having their access cut off to senior officials or being openly criticized in meetings. Doctors who had previously worked in the military gave examples of being deliberately humiliated by their senior officers. Another common experience was for senior officials to put pressure on a doctor by contacting their family members and asking them to intervene. Doctors that challenge the MoHS could also be accused of doing so only to support the opposition political party, thereby delegitimizing their issue and opening them to public attacks.

*“Because it’s a small country and we all know each other, people know your families, sometimes you can be meeting somebody and if you push too hard, I’m sure if you still continue the meeting you can achieve something, but if they feel pressure, then they’ll just think,’ Oh, well, I can call his uncle and push’.* Junior doctor, male

In one incident in 2021, several senior officials at the MoHS were alleged to have physically assaulted a junior doctor on hospital grounds for publicizing a protest action by cleaning staff. The doctor then faced multiple administrative charges by the MoHS and, following a sanctioned national strike of doctors organized in solidarity, a number of doctors reported having their salaries withheld.

While there was consensus that doctors feared retribution for challenging authority, some senior doctors argued that the risks were more perceived than real.

*“Some of these fears, sometimes they are more passive. They make them to look real, but sometimes if you stick on your grounds, it’s just rather vague threat or fear.”* Senior doctor male

Several participants also argued that doctors had a duty to work in rural and underserved communities and that while intended as a punishment, a posting there should be received as a responsibility.

*“We said look, wherever you are transferring us is in Sierra Leone, it is not a strange land. We are meant to work in Sierra Leone. So to hell with your transfer, we’re fighting for this.”* Senior doctor, male

Overall, many examples of retribution were given, demonstrating that these threats were, in fact, real. Senior doctors also noted that these mechanisms that officials used to silence doctors were not new but had been common practice for decades.

The language commonly used to describe the MoHS was also striking. One participant described senior officials as a ‘*cabal*’ while another said that the Ministry relied on ‘*godfatherism*’. They described a dynamic of underqualified people being appointed to senior posts in government, provided they demonstrated allegiance to their political patrons who put them there. Once in post, those unqualified officials would comply with any directive for fear of losing their jobs, knowing that they would struggle to find similar employment elsewhere. This was perceived to have created a patronage network that gives considerable power to the most senior officials to push through decisions that may be outside of bureaucratic norms.

Several participants also noted that these dynamics were not specific of the health system but could be seen across sectors in Sierra Leone. Participants also made comparisons with the public health sectors in Nigeria and Ghana, were some of these dynamics were perceived to still exist but to be less severe, such that it was significantly easier to lead within the system in those settings.

### 4.2 Exit from the public health system

The challenge of leadership was described as one of several contributing factors that push people to leave the government health sector, either to go abroad or to the non-government sector.

*“Sometimes some people are forced to leave because they have no choice, because they tried to make the change, and they see that they have already muddied the waters. And they know that ‘Well, you know, I’m not gonna make a move here’. There are other people who are just kind of like, who realize and are visionary and say that look, the only way I can make this change is getting out.”* Senior doctor, female

The issue of experiencing barriers to leading change was not generally considered the main cause of emigration by doctors, however; a range of other factors were also described as contributing to exit, such as low salaries and poor working environment. One participant did not consider barriers to leading change to be a cause of exit at all.

The emigration of doctors was considered to negatively impact the health sector, because it reduces the availability of qualified health workers, particularly those doctors who were leading efforts to make change. It was also seen to have financial implications because of the loss of the investment made by government to provide training scholarships.

*“In my time I’ve seen a lot of doctors leave, left country, who are potential leaders, people that can make change, and their colleagues look at them and say, wow, come back, if you stay you could do more. And I’ve seen a lot of them left.”* Junior doctor, male

There was consensus from participants that the health system was not sensitive to the exit of doctors and, therefore, exit as a personal strategy does not appear to currently create pressure for system change in Sierra Leone. People in authority ‘do not know and do not care’. In many cases, people in authority may be relieved when doctors who challenge the system leave, although most participants felt they were not deliberately being targeted to exit.

*“They leave. And it depends on how they leave. Many times people just get frustrated and say, you know what, I can’t change the system, or the organization is not good. Let me just go quietly, and they go quietly. And so because of that, maybe managements don’t realize that it is because of these reasons. Or maybe they are just complacent, as you say, not sensitive to the issues thinking that maybe people may be overblowing it because they’re unhappy with the* status quo*, you know. So, I think it has not yet come like in other societies, wherein if such a thing does happen, someone is there to follow through and say, ‘Hey, this person has been calling for these things, he or she has left, can we try to avoid that and put things in place?’ Many times, people are not aware of why people leave.”* Senior doctor, male

*“Nothing. No. Who is going to hold who to account? There will be absolutely nothing. In fact, excuse my French, some people will say, good riddance … Because they would have their way quicker, better and easier. Because that person may have been somebody that is obstructing, trying to put things right etc, and it doesn’t make a difference to them.”* Senior doctor, male

One important explanation for why senior officials were not more concerned about doctors leaving the government health system is that, as senior civil servants, they were able to obtain public funds to get medical care abroad or had the money to take their families to private facilities when they needed medical care. Consequently, the poor performance of the government health system did not affect them in a personal capacity.

*“They are very rich, they are going abroad, their kids are being educated abroad. When they get sick, they are flown abroad. We also work in private hospitals in our downtime, and you see that their kids are being seen in these private hospitals, they have health insurance that you don’t have.”* Middle-grade doctor female

There was only one clear example given of where the exit of doctors did result in system change, where an international donor supporting a particular health facility put pressure on the government to address the challenge that was driving doctors away.

Exit to the private sector was common and there were varied views on its impact. While many participants viewed this as a loss to the government health system, one private doctor felt they were better able to drive public sector change from the private sector because of the professional independence it enabled. They felt, however, that doctors in the private sector are largely cut out by the MoHS from opportunities to collaborate or access further training.

The role of the diaspora in affecting change from abroad was a focus of the interviews and was universally considered very limited. Participants felt that doctors who had moved abroad quickly ceased to understand the realities or political dynamics on the ground and lost the respect of their colleagues. A specific limited role for diaspora Sierra Leonean doctors to support with fundraising and engaging through organizations that were on the ground in the country was mentioned. There was also a view that doctors who did not depend on the Sierra Leone government health system for their livelihood could be more outspoken in raising important issues, but that too often diaspora doctors did not take this role.

*“And I think because I come from the outside, my livelihood is from the outside. I can come to Sierra Leone and shake things up because I don’t care, you’re not going to fire me … I’m at the point in my career where I just want to make things work. And I will tell you the truth. And I don’t have big consequences … I don’t have that fear and that stranglehold that’s stifling leadership at all levels.”* Senior doctor, female

While participants discussed a range of factors that were driving the exit of doctors from the public sector, a theme that was repeatedly mentioned as enabling exit was opportunity.

*“Well some leave because they have the opportunity of going out. Some decided to stay because they had no opportunity of going out, like, someone who doesn't know anyone in America who doesn't have any way or any relative who can help them. He only depends on scholarship. Yeah. So that person has to stay.”* Senior doctor, male

To emigrate required a set of professional and personal circumstances to be in place. Importantly, those with postgraduate clinical training were considered much better positioned to get jobs abroad than those who were medical officers or who were public health trained. Other factors included visas, funds and having family abroad who could assist with the relocation.

Leaving to the private sector also depended on opportunity, since in most cases this required doctors to open their own medical practices or clinics and therefore to rent property and buy specialist equipment.

### 4.3 Sticking it out – voice or neglect

When doctors faced barriers to leading change but stayed in the government system, they could either continue to exercise ‘voice’ and advocate for change or become complicit through ‘neglect’.

Few examples were given by participants of successful change being achieved by doctors who stayed in the system, and these were mostly driven by junior doctors. The general sentiment was that the pressure to conform was too great and that effective use of voice was impossible. Some senior doctors argued that positive change was possible but that it required persistence, diplomacy and seizing small opportunities as they emerged.

*“One or two militant junior doctors said no, we’re not putting up with this anymore. For example, when it came to doctors fighting for better conditions of service, better pay. I mean, they, when they, when was it about two, three years ago, they said right, we are going on a strike. And the Minister thought, you know, he’d use the same bully tactics. And he realized it didn’t work. Then he now had to come around the table.”* Senior doctor male

Notably, two doctors that described being able to achieve significant change with minimal resistance were based in rural districts, and they proposed that their work there faced less involvement from senior officials at the MoHS in Freetown.

*“So when I got to [rural hospital] … the hospital was in disarray, management wise, human resource wise, revenue generation wise, and, and even the quality of care wise … And one of the main, main issues was middle manpower, both for the clinician and the midwives. So we had to put a lot of pressure on the Ministry. And we got, that year we got like, [significant additional staff deployed].”* Middle-grade doctor, male

A repeated view was that if a doctor does not exit then they would inevitably succumb to pressures from the MoHS. Participants gave examples of doctors either becoming very quiet after experiencing retribution from their efforts of leaving change, or actively becoming complicit with the negative dynamics of the status quo (such as corruption).

*“Either they break you, and then you can continue to be in it, or they don’t break you and then you step out. And there’s always an attempt to break you. So there’s always going to be those tests to break you and there’s a lot of them and they come in different ways”* Junior doctor, male

*“If you’re in a toxic environment where everybody is looking at you, and you’re not in the mix … It becomes difficult. If you don’t leave, you join them, that’s for sure. If you do not leave, you will eventually become part of the problem.”* Junior doctor, female

There was however some optimism for generational change because of a new cohort of doctors returning from postgraduate training who were highly qualified and determined to make the system work. The view was that their qualifications would make it harder for the MoHS to repost them, while the threat of withholding scholarships had expired, and that a substantial number of doctors returning simultaneously might create critical mass for change.

*“It is quite heartening now to see some of them becoming very involved in in restructuring the system. And that is the only way the system will change. Because those who are in the system already are very happy, thank you very much, they got there through patronage or through whatever dark means, and they’re happy for things to stay the same. So, it is hoped that they will all die off, or retire, and these young ones will come in, who have been outside of Sierra Leone and seen the light and seen the way things are done”* Senior doctor, female

### 4.4 Contested loyalties

While Hirschman's theory refers to loyalty in the singular, participants of this study described a range of competing loyalties that shaped their decisions in different ways.

There were several loyalties that were considered to encourage doctors to stay in the government health system and not exit. The most frequently mentioned was loyalty to patients and poorer communities, which was coupled with feeling ashamed of the health system and wanting to change it. This commitment was particularly emphasized by two doctors who had trained at socialist medical schools in Latin America.

*“I think a lot of doctors want to stay, want to work and want to be part of the system because there’s a sense of pride that comes with working, especially when you work in the public sector and when you have contact with the vulnerable people. So I think there’s a sense of satisfaction that comes from helping people.”* Junior doctor, male

Another common loyalty was a sense of duty to stay after having received government training scholarships.

*“I got a scholarship from the government of Sierra Leone, basically from the people of Sierra Leone … And I felt obligated. And I’m sure there are others that feel obligated that you cannot use the money for country and then thereafter just move off and leave the people because I mean, they paid for you.”* Senior doctor, male

Participants described a loyalty to their professionalism as doctors, and to supporting their juniors. They also spoke of a feeling of patriotism, particularly those doctors who had done postgraduate training in Ghana or Nigeria, which appeared to have strengthened their feeling of pride to be Sierra Leonean and their motivation to bring the health system in their own country up to the standards of its neighbors. Finally, several participants described feeling loyalty to their families, both to stay in the country to be close to them but also to improve the health services they could access.

*“I think for some, for some, I think it’s the family there that they don’t want to leave behind. You know, it’s easier, you know, for Sierra Leone it’s more family needs than anything. So some people will be like, ‘I have my mom here, she’s old, I don’t want to leave her, I would prefer to stay.’”* Junior doctor, female

Noticeably, many participants explicitly mentioned that they did not feel loyalty to their government hospital, the MoHS or to the Government. Instead, doctors described loyalty as a two-way process, and asked how they could be expected to be loyal to the government when the government showed no loyalty to them. This negative sentiment towards government institutions and officials was instead a driver for them to exit. Connected to this, participants spoke of how they needed to show loyalty to themselves, including their values or faith, by exiting to somewhere they could progress professionally and make enough income to support their families.

*“I think we all start out with very deep loyalty to the government and to the people. I was always saying, I don’t want to set up a private clinic. I want to work in government because I am interested in pediatrics and a majority of the children are poor and coming to the government facilities. But they break, they crush that loyalty out of you … Who, who is supposed to ask me for that loyalty? Why should I put that particular kind of government ahead of my own family”* Middle grade doctor, female.

Participants often disagreed with each other on the role of these loyalties, while others noted that these loyalties were personal to the individual. While these competing loyalties influenced decisions to stay in the government health system or to exit, the decision to stay did not always result in the exercise in voice, although in some instances it clearly did.

*“I think it’s a personal thing. It’s really individual, some people feel loyalty to the nation. Some people feel loyalty to themselves, I need to feed my family, I need to put food on my table … Other people feel like I need to make a change in this country. I need to make, you know, make things happen. I need to make a difference”* Senior doctor, female

A final theme was the importance of not confusing loyalty with a lack of opportunity; if a doctor only stayed because they had no opportunity to leave then that was not loyalty, instead it was only those who stayed despite the opportunity to leave who could be considered loyal.

### 4.5 Exit or voice?

When asked which approach was more effective at triggering change in the government health system, to exit in protest or to use voice to speak out in protest, there was a unanimous view that exit abroad did not lead to change and few participants suggested that exit to the private sector could allow for impact in the public sector.

*“Well yeah, so, but for me I think we have to stay within a county and see whether we can improve it. Every day or every week I ask myself do I need to stay to do it or do I need to go to the private sector and do it? For-profit or not-for-profit? What way can I do it? But I know I’m doing it … So what I tell colleagues is, we cannot be out and change it”* Junior doctor, male

The views of participants on the relative effectiveness of using voice within the public sector, moving to the private sector, exit abroad, or continued activism in the diaspora, only weakly correlated with their own personal decisions about whether to leave or stay. While participants often did suggest that health system change might be achieved from the position in the system they had chosen to take, they were often quite open about the limitations and challenges of this or about what impact, if any, they had been able to achieve themselves.

While participants agreed that staying in the system was always preferable, many of them were also very pessimistic about whether voice worked, arguing that doctors were always broken by the system and would give up on voice in favor of neglect. Those participants, therefore, felt that change was impossible, although some had hope that a new generation of doctors might challenge this.

## 5 Discussion

This study shines a light on how commonly doctors in Sierra Leone experience retribution from their efforts to lead change and unearths a longstanding set of tactics that are used by those in power to achieve this. This retribution drove some doctors to leave the public sector while forcing others into silence or complicity, leaving few who were able or willing to continue use their voice to speak out for change. Our findings can be used to expand Hirschman's theory for the context of health workers leading change, generating a conceptual framework that can guide policymakers in how to both strengthen health leadership while also reducing the hemorrhaging of critically needed health workers from the public sector.

### 5.1 Expanding Hirschman's theory for leadership by doctors

**Fig. 1:**
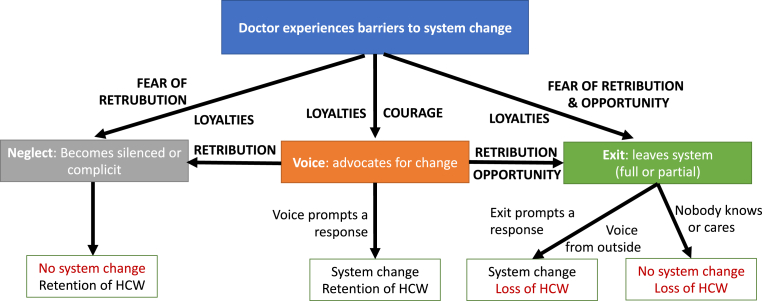
Expanded Hirschman's theory: An illustration of choices available to doctors experiencing barriers to health system change.

Across the interviews, a key theme was the complex field of pressures and factors that underpin doctor's career choices and circumscribe their efforts to lead change. Hirshmann's framework helped illuminate those barriers. The concepts of ‘exit’ and ‘voice’ were intuitive to participants and appeared to aid them in articulating their experiences. Our findings corroborate Rusbult et al. (1982) earlier addition to the theory of ‘neglect’ as a third outcome, with many examples given of doctors who decide to stay in the government health system but become quiet or complicit rather than continuing to utilize voice. Our work expands on Hirschman's theory in this context it in several ways, which are captured visually in Fig. 1.

Diaspora doctors were shown to play an on-going role in the country's health system; there is, however, no obvious place for this group in Hirschman's original theory, and we propose including a category of ‘partial exit’ to describe those who have left but continue to engage. This label could similarly be used to describe doctors who have stayed in the country but moved to the private sector, while continuing to actively work for change in the government health system. Our findings show that the effectiveness of doctors who ‘partially exit’ the system in Sierra Leone may currently be mixed, but participants also suggested that using voice from outside the system could have greater impact if more effectively utilized. Doctors who have partially exited continue to express forms of loyalty to the public health system or the communities it serves. A 2019 review identified 89 medical diaspora organizations in the USA, UK, Canada and Australia for professionals from low- and middle-income countries. However, advocacy for domestical health system reform was not included in the primary activities of those groups, suggesting our findings from Sierra Leone are relevant in other settings (Frehywot et al., 2019).

Also neglected in Hirschman's original theory is the importance of opportunity as a moderating factor for exit. Participants in our study emphasized that not all doctors have the opportunity to exit, based on a range of personal and external factors, and many argued that opportunity, for example sufficient finances and personal connections in other countries, was the most significant determinant of their decision to stay.

While Hirschman acknowledges that voice requires effort, his original work made little mention of the risks and negative consequences that may result from it. Thus, in addition to the empirical insights it provides into health system strengthening, this study offers theoretical dividends, pointing to the key importance of loyalty, which is notably underdeveloped aspect of Hirschman's theory. We found that doctors did not experience loyalty as a single phenomenon but rather as multiple competing loyalties. Where Hirschman proposes that loyalty, as a rule, “holds exit at bay and activates voice” we found that loyalties can be drivers for exit as well as voice (1970, p. 78).

### 5.2 Using an expanded Hirschman's theory as a framework for policymakers

This conceptual development of Hirshmann's theory can provide insights to policymakers who seek to retain doctors in the government health system while encouraging them to lead change. Our study documented several examples of leadership by doctors that aligned with health system strengthening goals, for example standing against corruption or developing specialist services. There were, however, also examples where their advocacy was primarily focused on the working conditions of doctors, such as increasing salaries. While these may sometimes contribute to the health system, for example by improving staff retention, they risk also shifting resources away from other priorities. Policymakers therefore need to find the right balance in supporting the advocacy efforts of doctors.

Exit could be reduced by addressing the retribution experienced by doctors trying to lead. In Sierra Leone, one example of this would be to reform the process around postings and awarding of scholarship by establishing a transparent system based on merit, and shifting authority for this to a committee that is more independent of MoHS politics. Another option, that could be driven by doctors themselves rather than government officials, would be to strengthen medical associations to be more proactive in defending doctors who are threatened with the withdrawal of salaries or other forms of intimidation.

Domestic policymakers have less control over the issue of opportunity, particularly when it comes to emigration, since this also depends on the policies of other countries; for example, one participant commented that changing immigration policies in Australia and the USA have reduced the opportunities for exit by Sierra Leonean doctors over the last twenty years. Countries that receive large numbers of foreign-trained doctors, such as the United Kingdom and USA, should re-enforce their commitment and adherence to the WHO Global Code of Practice on the International Recruitment of Health Personnel (2010).

Increasing the visibility of exit may help to address the situation where officials ‘do not know and do not care’ if a doctor leaves. A starting place would be to collect data on the causes and scale of exit by doctors and communicate this widely. Policy options include ensuring that officials experience the consequences of exit alongside other citizens, such as by withdrawing government funds to send MoHS officials overseas for medical treatment.

Efforts should also be made to increase the audibility of voice. By taking a more organized approach to advocacy, such as by forming associations, doctors can make collective expressions of voice, which may provide some protection from retribution. Mentoring and support could be provided to mid-level doctors who are fighting for change, to prevent them sliding towards neglect and to disrupt the current dynamic of ‘you don't change the system, the system changes you’. A review of promotions and postings to ensure that these are based on merit rather than political connections may increase the independence of officials and disrupt patronage networks. The overall objective should be a shift in culture, where leadership by doctors is viewed as an opportunity rather than a threat.

Finally, policymakers should be encouraged to understand loyalties and strengthen those that encourage doctors to stay, such as efforts to inspire greater commitment to patients and the profession in medical training, which was evident in the two participants who had trained at socialist medical schools in Latin American. Our research also shows the value in providing scholarships, which is reflected in other studies that show their association with retention and a sense of belonging (Ross et al., 2015; Schmiedeknecht et al., 2015). The relationship doctors have with the MoHS and their own hospitals may need to be reimagined to build more institutional loyalty, alongside a broader appeal to patriotism. Competing loyalties that are drivers for exit should be addressed, such as providing a salary that meets basic needs and a working environment where personal values are not compromised.

### 5.3 Health systems strengthening as a political as well as technical exercise

An important theme of this study is that the barriers to leadership in the health system were not only technical but also political. Participants described how advocating for change often threatened the self-interest of others, particularly senior officials, which led to retribution and an attempt to ‘break you’ into silence or complicity. This finding aligns with other studies, which have found, for example, that “across countries, the existing public sector context provides barriers to exercising … participatory leadership … Among these barriers are not only resource shortages, but also features of organizational culture” (Gilson & Agyepong, 2018, p. ii2).

By contrast, health systems strengthening initiatives undertaken by the global health community commonly focus on ‘capacity building’, which is defined in one WHO glossary as “the development of knowledge, skills, commitment, structures, systems and leadership” (Smith et al., 2006). A scoping review on leadership training programs for health professionals in Sub-Saharan Africa found a strong emphasis on technical skills and knowledge in many of the 27 curricula that were analyzed (Johnson et al., 2021). Informed by Hirschman's theory, this study would suggest the importance of a more sociological approach to health systems strengthening, recognizing the individual agency of health workers and the power dynamics at the organizational and societal level that shape how health systems function, and indeed contributing to the conceptualization of what a system is Herrick & Brooks' (2018) research on global health partnerships generated similar findings, commenting that “The overriding biomedical evaluative frame of quantification, metrics, indicators, and outcome measures questioned by some stands in stark contrast to the everyday human performances that come to shape partnership working, including those that lead to varying degrees of efficacy in the multifarious (and hugely political) contexts of [health system strengthening] and [human resources for health]”.

While our work encourages the recognition of health system reform as a political exercise, it does not invalidate a role also for technical initiatives to create a more enabling environment for leadership and change. For example, supporting health workers to strengthen their professional qualifications, such as postgraduate clinical training, was found in our research to confer status and job stability, and therefore empower them as political actors. Similarly, formal leadership training within health professions education may equip graduates with skills to navigate political dynamics, to mobilize people for change, and foster networks with peers and seniors that can again be protective against retribution (Doherty et al., 2018; Kebede et al., 2012; Mutale et al., 2017).

While global health actors may try to be impartial, they must recognize that they will never be neutral, because by giving control over programs and funding to those in authority, they are directly influencing those power dynamics and are therefore ‘picking sides’ in continuous negotiations between different parties in a way that may ultimately undermine efforts to achieve change. An alternative approach would start with global health actors seeking to better understand the power dynamics at play in a system. Hirschman's framework can assist with this by acting as a guide. This would enable reflection on how programs impact power dynamics within the system and ultimately system change. Such an approach might seek balance in power between key stakeholders, for example by also centering health professionals and professional associations in collaborations, alongside support to government officials.

### 5.4 Strengths and limitations

Exclusively qualitative, this study does not seek to make generalized pronouncements on the Sierra Leone's health workforce, let alone the experience of all African countries. The focus specifically on doctors, moreover, excludes the potential role other stakeholders in the health system, such as policy makers and patient-publics who would have helped deepen and potentially provide a contrasting perspective on health leadership developed here. The positionality of the lead researcher, as a non-Sierra Leonean, and having existing relationships with several interviewees, may have introduced bias into the participant comments and the data analysis. That being said, we feel the thematic insights generated hold relevance for policy makers on the choices available to doctors facing retribution to leading change and the factors that shape their decisions.

### 5.5 Further research

Our study unearths a number of areas where further research is needed to enable a fuller understanding of the drivers of barriers to health workers leading change and their consequences. Our qualitative work would be well complemented by the collection of quantitative data on health worker exit from the health system, both to the private sector and abroad, to illustrate the scale of this problem. This research should further seek to understand who these professionals are that exit, for example their previous or intended roles in driving change, rather than assume that all professionals play the same role in any health system.

A historical analysis of successful reform efforts in the health system would also help to explain positively where and how change has been successful, recognizing that reform is a complex, challenging and slow process. Our study should also be replicated with other professional cadres and in other countries to explore the extent to which the dynamics we describe are particular to Sierra Leone or are more widely generalizable. Finally, testing the utility of our expanded Hirschman framework for policymakers trying to encourage system change and reduce health worker attrition would illustrate its potential value in reform efforts.

## 6 Conclusion

This study sought to explore how best to support the practice of leadership among doctors by drawing on Hirschman's theory of ‘Exit, Voice & Loyalty’ to examine how doctors in Sierra Leone perceived the barriers they encounter from trying to lead. We found that doctors can experience many forms of negative personal and professional consequences from attempting to change the health system, and that this retribution is a driver of doctors becoming silenced, or exiting the government sector by moving abroad or to private employment. We further build on Hirschman's theory to provide an expanded conceptual framework that includes several additional components, including the option of ‘neglect’ for those that stay in the government sector but become silenced or complicit, the role of opportunity in decisions to exit the system, and the existence of ‘partial exit’ for those that continue try drive change from the private sector or diaspora. This expanded framework can be used by policymakers to identify opportunities to improve health worker retention and support doctor-led change. Importantly, the study highlights the need for global health actors to develop a more sociological lens to health system strengthening efforts and recognize the impact that their work has, intentionally or not, on the power dynamics that shape efforts at reform. Further research is needed to show the scale of health worker exit to the private sector and abroad in Sierra Leone, to examine whether these findings are reflected for other professional cadres and in other countries, and to test whether this expanded framework is useful as a guide for policymakers.

## Funding

NS, AHK and OJ are supported by the 10.13039/501100000272National Institute of Health Research (NIHR) Global Health Research Unit on Health System Strengthening in Sub-Saharan Africa, King's College London (GHRU 16/136/54) using UK aid from the UK Government to support global health research. The views expressed in this publication are those of the author(s) and not necessarily those of the NIHR or the Department of Health and Social Care. NS′ research is supported by the 10.13039/501100019219National Institute for Health Research (NIHR) Applied Research Collaboration (ARC) South London at King's College Hospital NHS Foundation Trust. NS is a member of King's Improvement Science, which offers co-funding to the NIHR ARC South London and is funded by 10.13039/501100002102King's Health Partners (Guy's and St Thomas' NHS Foundation Trust, King's College Hospital NHS Foundation Trust, King's College London and South London and Maudsley NHS Foundation Trust), and the Guy's and St Thomas' Foundation. NS′ research is further supported by the ASPIRES research programme (Antibiotic use across Surgical Pathways - Investigating, Redesigning and Evaluating Systems), funded by the 10.13039/501100000269Economic and Social Research Council. AHK also receives support from the 10.13039/100010663European Research Council (ERC) under the European Union's Horizon 2020 research and innovation programme, investigating the Design and Use of Diagnostic Devices in Global Health (DiaDev), under grant agreement No 715450. Finally, she receives support from MRC-AHRC as part of their GCRF programme for HAPPEE, a project on preventing pre-eclampsia complications through community engagement and education, (ref: MC_PC_MR/R024510/1).

## Ethical approval

The study received ethical approval from both the Psychiatry, Nursing and Midwifery Research Ethics Subcommittee at King's College London, UK (LRS-18/19–10994) and the Sierra Leone Ethics & Scientific Review Committee.

## Declaration of competing interest

The authors declare the following financial interests/personal relationships which may be considered as potential competing interests:

NS is the director of the London Safety and Training Solutions Ltd, which offers training in patient safety, implementation solutions and human factors to healthcare organizations and the pharmaceutical industry. The other authors have no conflicts of interest to declare.
