# Supplementary material for: Exit, voice or neglect: Understanding the choices faced by doctors experiencing barriers to leading health system change through the case of Sierra Leone
Source: SSM Qual Res Health. 2022 Dec;2:None. doi: 10.1016/j.ssmqr.2022.100123 (PMC9748299; doi:10.1016/j.ssmqr.2022.100123)
Supplement: Multimedia component 1 [file mmc1.docx]

**Supplementary Appendix A: Interview Topic Guide**

**Background to Participant [if not included in phase 1]**

1. To start off, I wanted to ask a bit about your own career. Can you very briefly give me a sense of your own background and training, as well as your current role now?

**Background to the Project**

- This is the second phase of a research study looking at leadership by doctors in Sierra Leone
- In the first phase, we did fifteen interviews and our three key findings are summarised in this powerpoint.

1. What did you think about the themes that emerged from our earlier study – do they resonate with you?
   1. Is there anything in particular that you would add or view differently?

**Background to Leadership Challenges**

- In this interview I want to explore in more detail the leadership challenges that doctors in Sierra Leone experience.

1. Can you give any specific examples that you’ve experienced yourself, or observerd with others, where doctors have faced challenges when trying to practice effective leadership in Sierra Leone?
   1. What were those challenges?
   2. Why are those challenges there?
   3. How did you or the person observed respond to these challenges?
2. Do doctors ever face negative consequences on their jobs or careers from trying to practice effective leadership?
   1. Can you give any examples?

**Leadership & Exit**

1. In previous interviews it was suggested that some people leave the country or the government health sector because of the push back they received in trying to exercise leadership. What do you think about this?
   1. Can you think of any examples where doctors (prompt ‘which might include you’ if interviewee is abroad’) have decided to leave government service or move abroad as a result of this?
2. [If yes to 5] What impact does their leaving have on the health system?
   1. Prompt: does it impact on patients or communities?
   2. If doctors leave, does it have any specific impacts on people who have authority over the government health system, such as politicians or Ministry of Health & Sanitation officials?

**Leadership & Voice**

1. Those doctors that experience leadership challenges or the negative consequences of it, can you think of any examples of where they decided to stay in the government system and advocate for change?
2. [If yes] Was their advocacy successful, can you think of any examples where they achieved positive change?
3. What impacts, if any, did their decision to stay and advocate for change have on them as individuals?

**Leadership & Commitment to Stay**

1. Why do you think some doctors who experience leadership challenges choose to leave while others choose to stay?
2. Which do you think is a more effective approach to bringing about change in the health system in Sierra Leone, leaving in protest or staying and advocating from within?
3. Do doctors feel a particular loyalty to to the health system in Sierra Leone?
   1. What shapes that sense of loyalty, what are people feeling loyal to in particular?
   2. How does that sense of loyalty impact on their decisions to leave or stay when facing leadership challenges?
   3. Do doctors in different parts of the health system – such as in the military, in clinical practice or in the provinces – experience loyalty differently from others?
