# Supplementary material for: Exit, voice or neglect: Understanding the choices faced by doctors experiencing barriers to leading health system change through the case of Sierra Leone
Source: SSM Qual Res Health. 2022 Dec;2:None. doi: 10.1016/j.ssmqr.2022.100123 (PMC9748299; doi:10.1016/j.ssmqr.2022.100123)
Supplement: Multimedia component 2 [file mmc2.docx]

**Supplementary Appendix B: Additional Qualitative Data**

| 1. ***Retribution as Tradition*** | | |
| --- | --- | --- |
| Reposting to remote or undesirable locations | | *“I knew a doctor who was sent to the provinces because of strike action, he was immediately taken from Freetown sent directly to the provinces because of this… He is still in the provinces so far as I’m aware. So these are why people are scared.”* Junior doctor, female  *“So it’s, it’s quite challenging and even within [tertiary hospital], those who may not comply by the ‘rules’, because at the end of the day, they will be posted to the outpatient department permanently and that’s where the heavy crowd and the heavy work is.”* Senior doctor, male |
| Withholding of salaries | | *“He was having a hard time. Sometimes he would get a phone call from the Ministry ‘We are going to stop your salary if you post [on social media] again, if you say this again, if you take this interview again.”* Junior doctor, male |
| Career stalled or losing job | | *“It’s, it’s serious consequences. It’s, you know, being demoted, losing your job”* Senior doctor, female  *“I remember when we started the strike last year, and there were threats from our senior colleagues saying ‘You have to stop this, if you don't stop this then you will repeat your postings’”* Junior doctor, male  *“When you fall, when you fall into the bad books of the hierarchy, then it’s like, it’s like, you have a big brick wall in front of you.”* Junior doctor, male  *“I had a friend that came that was leading one of the hospitals, and he was introducing some principles and things. And so the next thing is they set off a fire in the hospital. And then they blamed him and so as to be sacked…. He had to lose his position.”* Junior doctor, male |
| Withdrawal of scholarship | | *“I know of colleagues, or young doctors, who have applied for a scholarship and at the end of the day are refused simply because they just do not comply by the ‘rules’.”* Senior doctor, male  *“Some of us had to be victimised. I remember a colleague was denied a scholarship that he won. He had to reinterview and he was actual told he was not going to be interviewed for that particular scholarship!”* Junior doctor, male |
| Cold-shouldered by Ministry | | *“By the time I got to the director, he was already on edge, [he said] ‘Oh you, you are this disrespectful guy, that went to the Office of the [directorate]. And so for that reason, I’m not even going to talk to you’… They even called one of my bosses that ‘Oh, you have to call your boy and warn him on this.’”* Middle-grade doctor, male |
| Humiliation | | *“[Senior military doctors] did all sorts of nasty things to us, humiliate us, make us march in front of privates or the lower rank officers, embarrass us openly, like some kind of punishment, embarrassing us every two or three weeks.”* Senior doctor, male |
| Public attacks, including accusations of being political | | *“Obviously, when it comes to more political issues, you could also be attacked by the public. So for example, when they heard about the advocacy we were doing that people will tell us and say ‘Oh, you’re from the opposition.’… Generally, if you are probably trying to advocate for anything, you write, even people who write on social media there will be attacked.”* Junior doctor, male |
| 1. ***Exit from the public health system.*** | | |
| Challenge of leadership as a contributing factor for exit | | *“We have this group of people who are [specialists] in Ghana, living their best life, being well paid. And they can just work without all of the politics and all of the fear… and they say, ‘You know, it’s not just about money, I will take a lot less to be able to be home and work. But it’s about this fear of, you can build something, and it can just be snatched away from you quickly.’”* Senior doctor, female  *“If you interview doctors in in from Sierra Leone, who are currently in England or America, they will tell you similar stories, all right… Because of this, the threats and the rural postings, and so on and so forth. Some of them, their parents could afford sending them abroad, so they left the system. So that is a key, a key factor. One of the key factors of them leaving, I’m not saying it is the main factor, but is one of the contributing factors”* Middle-grade doctor male  *“Nah, I really don’t think it’s got to do with push back in leadership issues. It’s, it’s if you want to go better yourself, if you want to go earn more money. That is why…”* Senior doctor, female. |
| Impacts of doctors leaving on the government health system | | *“It affects the health system negatively, you know, in terms of the numbers, or the health worker population density, you know, is affected by that*.” Middle-grade doctor, male  *“It’s actually affecting the economy. Because, okay, I usually tell them that with the specialists, the government will spend between $50,000 to $100,000, to take a single specialist abroad, I mean, in the West African sub region. So if the person comes back, and you allow that person because of your own personal issues, your own fear, and that person leaves the health system, that is a huge economic loss to the government”* Middle-grade doctor, male |
| No sensitivity of the health system to exit | | *“The issue is whether… well how many people know and how many people care? You understand? You come among your few friends and the rest, you grumble a bit, and you leave, you know? It’s not new. It’s not new at all. It’s something that happened in a generation before us and before the last.”* Senior doctor, male  *“There’s no consequences for that, because they don’t care. Well, for me, when we understand that our leaders in the Ministry of Health really do not care, then we will understand a lot of these things that they do and why the health system is not working. They don’t care that we leave, they don’t care that we don’t have enough doctors, deep down they don’t care. Even if they think ‘All the doctors are leaving’ they really do not care.”* Middle-grade doctor, female |
| Explanations for why the Ministry is not sensitive to exit | | *“You find the guys who are the Ministry, and we’re talking about the guys in the Ministry here. They don’t care. They they’re not clinicians, they don’t care, no matter what we complain about, they don’t care how things work. They know that if they’re ill they get boarded, they go off to India or Ghana or wherever it is they go now. So they just don’t care. You know?”* Senior doctor, female |
| Example of when the health system was sensitive to exit | | “*I was the first one that left and my other colleagues also, all four of us left. It affected them. The [international donor] went to them and said where are your doctors, we really like them? You let them leave because you suppressed them. You know, give them what they want. Since then, you can imagine the new people they recruited after a couple of years now, they are giving them scholarship to study [abroad]... [The facility leadership] were pressured and some of them were sacked*.” Senior doctor, male |
| Exit to private sector | | *“I know a number of people who, because they had problems with the Ministry of Health, with colleagues or with superiors, you know, so they just decided to go private… I think they would create more impact of the Ministry than in the private sector”* Senior doctor, male  *“Because there’s so much politics in the [government health] system, you have to move out… I would never been able to get people to do [training opportunity] in Sierra Leone, if I was in the government system. That was the only way I was able to do it, because I went, did this stuff myself.”* Senior doctor, female |
| Role of diaspora | | *“Well, in terms of actually a system change, it’s difficult for them to actually influence system change. Yes, they can come and do charity work, help with some activities, but in terms of actually changing the structure of the system. It’s difficult for them. It stems from two factors. One is that even for us, the local guys, the guys that remain in the country, we see that we don’t see them as patrons, alright? We see them as people, actually, due to the fact that the time most of them left Sierra Leone… we see them as people who actually abandoned their calling. Alright. So because of that, it’s difficult even when they come to talk to us about leadership and everything. It’s difficult for us to even listen to them… The second thing is that they’ve lost contact with context. All right, the fact that yes, you were born and went to school in Sierra Leone, you grew up to Sierra Leone, before you left, went to university. That doesn’t mean that you knows the now Sierra Leone... You know, so because of that loss of contact, they don’t understand the current process. So when they come in, when you try to come back and contribute, it is very difficult, some of them have attempted to come and join the system but they leave, because they could not, they could not cope with the current context of our society”* Middle-grade doctor, male  *“I think that is possible. And I think in fact, it’s probably one of the easiest ways to influence positive change within the healthcare delivery system in Sierra Leone but unfortunately I want to disagree with you that they are vocal, they are not. They are very, very passive, very, very quiet. When you look at the diaspora pressure on other issues as compared to health, diaspora, medical colleagues and practitioners have been very, very passive. There are many ways they could have influenced things.”* Senior doctor, male |
| 1. ***Opportunity to Exit*** | | |
| Opportunity to exit | | *“I left because I got the opportunity, my brother was here [abroad]. If I wouldn’t have had the opportunity, I don’t think it would have happened in any way, I don’t I come from a rich family, and I don’t have anybody in West Africa at that time… To go and study in West Africa is about $10,000, but I didn’t even have $2,000. So If my brother was not here, I don’t think I would have. So one of the reasons why people leave is because they have the opportunity, their family have got the money for them to go do postgraduate, and they are ambitious, and the family has got the money or they are outside in America, England. But if you come from a poor family and you don’t have those opportunities, you stay.”* Senior doctor, male  *“But you have to have the means to get out. If you don’t have the means, whether you have your great vision, whether you’ve muddied the waters, if you don’t have the means, and you have to put food on your table, again as I say, you’re just gonna stick where you are, and just try to hope for the best.”* Senior doctor, female  *“Not everybody has the means to run a private practice. So some people are like, I want to change but I just have to stop here. I don’t have the means or the capital to start private practice”* Senior doctor, female |
| 1. ***Sticking it Out – Voice or Neglect*** | | |
| Examples of voice | | *“Over the years, if I go back retrospect to now, I think one of the things one of the things that I’ve learned is if you want to change, stay in and you can change things, you may not be able to do the dramatic change automatically, but you may be able to influence if not all but certain percentage of the change.”* Senior doctor, male |
| Different experiences leading change in urban compared to rural areas | | *“It’s different yeah. Freetown is more challenging. In [rural district] while you might be challenged in terms of getting resources, like financial resources… you have staff that are well dedicated, staff that might not argue with you much, staff that will follow your instructions. Whereas at [urban hospital], you might have colleagues, doctors, that will not listen to you, because they think they are the same doctors, you all went through the same… And in Freetown, everyone is exposed to money, everyone at those big hospitals, what they want, from the nurses to the cleaners… everybody's just focused on money, not actually the service.”* Senior doctor, male |
| Voice getting broken to become neglect | | *“I was like ‘You were this straight guy, and always going for the right thing’. And he said, and this is a very successful person in the Ministry now, and he said ‘I think in this place, I really think for you to really get to the top, you can’t really be that straight. The system will never allow you.’*” Junior doctor, male  *“That is what we’ve got, it is actually learned helplessness. Because, because, I mean, they’ll give you maybe like a bit of a side comment on the side, but nobody wants to stick their head out anymore, you know? And that’s the idea of ‘This is how we know Sierra Leone to be, it’s never going to change. Why are you wasting your time? We just need to survive now’ You know? And you see it. I mean, people I least expected to be taking money from patients and they’re now literally just to survive they’re taking money from patients you know.”* Junior doctor, female  *“You’ll see them coming young and youthful, and with ideas. And over time, they realize it’s not going anywhere. So they just kind of exist… They find a way to shape you to conform and they suck the leadership out of you. And if I hadn’t seen it, I wouldn’t have believed that it was possible. But everyone knows.”* Senior doctor, female  *“I tried thinking of the ones who had been fighting, even before I came, to when I was in medical school. So some of them have left. Some of them now work in private. Some of them are now very quiet. For me, that’s it. I remember there’s a doctor who was very, very vocal. He was sent to the provinces. And now he’s just so quiet.”* Middle-grade doctor, female  *“There are some who remain, who stayed in the system, what happened was, instead of them making change happen, they were changed. I saw the system has a way of bringing the best and the worst out of you. So in most situations, what happens is that they use some mechanisms to, to force you to change.”* Middle-grade doctor, male  *“If you’re in a toxic environment where everybody is looking at you, and you’re not in the mix, you know, you’re not in the mix with them. You’re not joining them… It becomes difficult. If you don’t leave, you join them, that’s for sure. If you do not leave, you will eventually become part of the problem. I can guarantee you that there are many people whom I swore on my mother, on my father’s grave, were never going to be part of the problem and are now part of the problem. So it’s these little seeds of disenchantment, discontentment with the system and the inability to do anything, because you are trying and everybody’s knocking you down.”* Junior doctor, female |
| 1. ***Contested Loyalties*** | | |
| Loyalty to patients and poorer communities | | *“But there are others that think if you don’t change Sierra Leone, who do we expect to change Sierra Leone? We don’t expect people to always come in from their own country to change your own country without you put in any effort. So I think these are some of the issues that encouraged people to stay at home and move forward.”* Senior doctor, male  *“When we were in Nigeria, we admired Nigerians so much, because they were able to, with all the challenges, their challenges are no different from ours, but they had a system, they had a training program, they had a very nice, good set up, and all those things. And in the evening hours every day, we’ll sit down and discuss what changes we need to bring back home to contribute to our system. So we have that sense of responsibility that know we have to do something.”* Middle-grade doctor, male  *“We actually trained [in Latin America] under socialism, that you have to actually be dedicated to what you do you need to provide service for the community, you need to serve the people… When I finished in [Latin America], I had opportunities in staying, my sister was in the [Europe]. She said come and sit your [European medical] exam. I said, ‘No, let me come home.’ And, actually, I don't regret coming home, because this is what I have wanted, saving the lives of our people.”* Senior doctor, male |
| Loyalty from scholarships | | *“Since I started school, in Sierra Leone, I’ve always be having scholarships. So from class one to my [secondary school exams] I had scholarship, I entered university on scholarship, I went for postgraduate scholarship. So I see it personally, as a sense of, of responsibility for me to give back to the country.”* Middle-grade doctor, male |
| Loyalty to profession | | *“When you talk about loyalty, it is just this strong feeling, okay, of allegiance, of support, of allegiance to, to whatever you are doing, okay… I’m a loyal to my profession as a medical doctor, because I took an oath to practice as a doctor, and I wake up in the morning, and I say to myself, yes, you need to keep going to save lives.”* Senior doctor, male |
| Loyalty to juniors | | *“It is the love for the younger ones… Most of the young doctors are my students. I taught them in med school. And I see it also as the responsibility that I need to get my product to the endpoint where I want them to be better than… Alright? So when you look at them, I feel like ‘Oh, if I, if I leave now it is like I’ve abandoned them’. When I come back what I say to them, the system is still the same. So those are some of the reasons why that small cohort of us decided to stay and work.”* Middle-grade doctor, male |
| Loyalty to country, patriotism | | *“It was loyalty to my country. Period. My wife was with me, we could started a family… It was not that I owed a loyalty to a particular hospital, or I owed a loyalty to a colleague, no, it was just to the country that hey, I think my country needs me. I’ve been there during the war. Now the war is over I just need to come back.”* Senior doctor, male |
| Loyalty to family | | *“The younger ones that are thinking that yes, the grass is not always greener when you go outside, you’re leaving your family at home and home is always home. And they want to stay home. And they don’t see why they have to be hastened out of the country...* *Especially because they are all getting married early. They are having kids early. So that is forcing them I think more and more to stay. So therefore they are now pushing for improvements. So that their staying is good for them.”* Senior doctor, female |
| Loyalty to government institutions and officials | | *“When we came back…we had so many challenges, and there were times when we thought that our loyalty was not being appreciated by our so called seniors by then. And there was a time for example, I thought that look, it’s rather unfair for me to stay in the system, and I almost left for the States, simply because my loyalty was not appreciated by my seniors… Because the loyalty stems from the fact that in as much as you may want to give service to your institution, or to the county in which you are working, but are they also loyal in what they are doing?”* Senior doctor, male  *“I give service more to my profession than that of the institution. The institution, my institution is [government] hospital, okay. I won’t say I am loyal to [government] hospital. Why should I be loyal to [government] hospital, okay? If I say I’m loyal to [government] hospital then, I would be dishonest with you.”* Senior doctor, male  *“To a great extent, I feel loyalty to the Ministry. And, you know, probably that’s, that’s, that’s because of my personal drive. The drive to make a difference. You know, and the very common one is the fact that you have, you have job security with the Ministry… For the first time ever, I got called I got a call on Christmas Day. And that call was from one of the Directors in the Ministry. And he actually called to check on me and to applaud me for the good work, you know, we’ve been doing in the district. And it felt very special, you know, and, and, you know, that alone, that in itself is a form of motivation.”* Middle grade doctor, male |
| Loyalty to self, including career, values and finances | | *“There is no sense of loyalty to your work, to the country to any of that, because every human being, your first sense of loyalty is to yourself and your family. And you need those needs met and when they are met, then you are free to do other things. But the system in Sierra Leone, those needs are not met. You know, you’re not paid enough. Your situation is very tenuous, where it’s linked to, at any point, you could lose it all.”* Senior doctor, female  *“I think a lot of it in Sierra Leone, in a lot of times, people tend to want to think about self, rather than as the greater good. And not because they’re selfish. Sometimes it’s just the fact it’s just the hard fact that I have to feed, thinking of the greater good is not going to feed my family. So people sometimes are forced to do that.”* Senior doctor, female  *“I always remember that was one of the reasons I could not sit there. Something was wrong. That’s just probably because of our personality and my personality. So that’s one of the things because I wasn’t going to be put in a position where I needed to kind of like, have to walk that thing. Um, and I guess it’s just the way I think. And so that was why I was like, get out of this, get out of where I have to be under the cloak of I can do what I believe is right.”* Senior doctor, female |
| Loyalty vs Opportunity | | *“So if I can’t talk about doctors generally being loyal, I see it as they are in a situation wherein they don’t have alternatives. So they are just coping with the situation. Alright, but there are some who are actually loyal to the system. When you now talk about loyalty is when you see this doctor with all the different opportunities lined up, and the doctor still decides to stay and work in the system. So if we want to assess the loyalty of the Sierra Leonean doctor, we have to provide him with all the other opportunities and see if he will choose Sierra Leone.”* Middle-grade doctor, male |
| 1. ***Exit or Voice*** | | |
|  | *“Oh my gosh, that’s so hard. Erm… My heart will say, my heart will say staying and fighting more effective. But then you have your family to feed, you want to progress, you want things in life or perhaps you just wants to, I don’t know, live a better life. And then your heart is saying one thing, but your head is saying something else…So if you had the numbers, we could stay on fight. That’s the heart talking. But then your head is logical and is saying, dude, if you want to survive, you had better leave, you know? I know. It sounds really horrible. But it is what it is.”* Junior doctor, female  *“I think to stay in fight, but it is, the people that I watched stay and fight is, you have to figure out how to navigate to best make change, right? It’s not straightforward. But there are, there are nuggets of light, and nuggets of change that you can get.”* Senior doctor, female  *“We just talk and talk and talk about change. And then after three, four years of no change, you’re like, oh, there’s no change. Let me go to plan B. And then we have a new crop of optimistic doctors who are like all that needs to be changed and changed and changed… That’s it.”* Middle-grade doctor, female | |
